# Factors contributing to routine immunization performance in Ethiopia, 2014

**DOI:** 10.11604/pamj.supp.2017.27.2.10470

**Published:** 2017-06-09

**Authors:** Aschalew Teka Bekele, Braka Fiona, Karengera Thomas, Aron Kassahun, Gallagher Kathleen, Peter Nsubuga, Yohannes Ababu, Assefu Lemlem

**Affiliations:** 1 Immunization programme, World Health Organization, Addis Ababa, Ethiopia; 2 Medical Epidemiologist, Global Public Health Solutions LLC, Decatur GA, USA

**Keywords:** Ethiopia, Immunization performance, disparity, immunization coverage

## Abstract

**Introduction:**

An increasing trend of routine immunization performance has generally been observed over the past decade in Ethiopia. However, the improvement is irregular with wide disparity among and within regions. This study analyzes health facility characteristics contribution to immunization performance in Ethiopia.

**Methods:**

We conducted a cross-sectional study and compared characteristics of health facilities in good and poor performing zones. We used administrative coverage reports and Personal Digital Assisted (PDA) supervisory data collected by WHO EPI field officers using a standardized structured checklist. We selected 48 zones and 302 health facilities based on immunization performance data and supervisory data on potential variables.

**Results:**

Logistics regression was used to identify independent contributors to good immunization performance. On logistics regression we found that: actions by higher levels in conducting supervision (Odds Ratio (OR) =4.15. 95% Confidence Interval (CI) = 1.85, 9.32, p value <0.01] and providing written feedback (Odds Ratio (OR) =4.35. 95% Confidence Interval (CI) = 2.27, 8.33, p value <0.01) , and provision of immunization services by the health facility itself for catchment population under each health unit (Odds Ratio (OR) =20.15. 95% Confidence Interval (CI) = 2.24, 181.38, p value =0.01) and absence of stock out of vaccines (Odds Ratio (OR) =0.44. 95% Confidence Interval (CI) = 0.23, 0.83, p value =0.01) are the likely significant factors contributing to good immunization performance in Ethiopia.

**Conclusion:**

Ensuring availability of immunization services in all health facilities, regular supervision and written feedback and improved stock management are essential factors contributing to good immunization performance.

## Introduction

Immunization is one of the most effective public health interventions known so far [1, 2]. In 2013, an estimated 2 to 3 million deaths globally were averted through immunization [3]. The Government of Ethiopia incorporated childhood vaccination in the Health Sector Development Plan (HSDP) as one of the key interventions to improve child health outcomes [4]. Progress was measured by proportion of under 1 year old children vaccinated with a third dose of diphtheria, pertussis and tetanus containing vaccine (DPT3) coverage which is the reference indicator of coverage for Expanded Programme on Immunization (EPI) [4, 5]. Ethiopia adopted the Global Vaccine Action Plan (GVAP) targets to reach 90% DPT3 coverage nationally and 80% coverage at district level by 2015 [6]. Between 2004 and 2014 improvement in immunization coverage was observed globally and in Africa [7]. The DPT3 administrative coverage in Ethiopia increased reports from 66% to 87% in 2004 and 2014 respectively [8].

Based on administrative and World Health Organization (WHO)-United Nations Children's Fund (UNICEF) joint estimates, immunization performance in Ethiopia showed improvement from 2012-2014. However, variation and discrepancies within regions and zones still remained a challenge where in 2013 and 2014 the proportion of zones that achieved DPT3 vaccination coverage of at least 80% were 50% and 73% respectively [8]. To improve and sustain routine immunization (RI) coverage, woredas (which are equivalent to districts in Ethiopia) need a supportive political and policy context, as well as essential immunization infrastructure [9]. The success factors for the improved coverage in Africa as well as Ethiopia were attributed to implementation of the “Reach Every District” (RED) approach, support of global partners, use of community health workers, and expansion of the health infrastructure and the health system [9–11]. There is little published evidence of studies conducted in Africa to identify the reasons why some jurisdictions perform well in immunization activities while others do not despite being in the same country setting of infrastructure and socio-economic contexts [12]. We performed a cross-sectional study to compare good and poor performing zones based on the characteristics of immunization programme management by health facilities to identify possible contributing factors to RI improvement in Ethiopia. We believe that the study results and recommendations could be used to sustain improvement in good performing zones and scale up good practices in poor performing zones.

## Methods

**Study area and study period:** Ethiopia is a country with a federal government composed of nine regional states and two city administrations. Regions are further sub-divided into 103 zones and 840 woredas which are equivalent to districts. As of 2014 the total population was estimated to be 89,714,313 with more than 3 million birth cohort targeted for immunization. Oromia, Amhara, Southern Nations and Nationalities People Region (SNNPR) and Tigray regions constitute more than 85% of the total population. There are over 3200 health centers and 15000 health posts and more than 90% of them provide immunization services. We collected data in July 2014 from 426 health facilities located in 53 zones to describe factors associated with good immunization performance in Ethiopia.

**Study design:** we conducted a cross-sectional study and compared characteristics of health facilities in good and poor performing zones to identify potential factors that contribute to immunization performance in Ethiopia.

**Figure 1:**
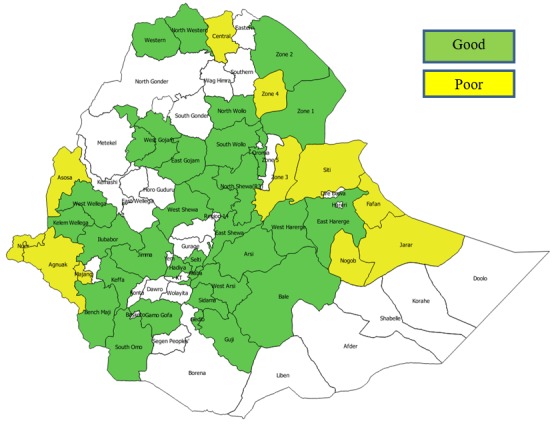
Map of good and poor performing zones in Ethiopia, 2014

**Study population:** we included 53 good and poor performing zones and studied characteristics of 426 health facilities that had data collected on the nine explanatory variables of this study: immunization service, session interruption, supervision, written feedback, defaulter tracking system, performance monitoring, vaccine stock status, availability of trained focal person and practice of informing care takers on key immunization messages. Health facilities from Harari region and Dire Dawa and Addis Ababa city administrations were not included as there was no supervisory data from these regions (Figure 1).

**Inclusion criteria:** we included zones with DPT3 coverage data for 2014 and health facilities with records on the nine independent variables.

**Sample size:** we selected 426 health facilities which had data on the nine explanatory variables and with available coverage data on third dose DPT3 coverage for their respective zones.

### Data collection and measurement

**Data source:** we used the joint Federal Ministry of Health - WHO-UNICEF report for administrative (JRF 2014) and used supervision data stored in the WHO database for analysis of health facility characteristics. The supervisory data were collected during health facility visits by WHO EPI field officers using a standard checklist adapted by the central EPI team uploaded in a PDA. The checklist contained questions on surveillance and immunization activities. Data were submitted to the regional and central coordinators and cleaned by central data management team.

### Study variables

**Dependent variable:** zone immunization coverage of third dose of diphtheria, pertussis and tetanus containing vaccines (DPT 3 coverage)

Independent variables: health facilities providing immunization service; health facilities that were supervised by higher level in the past 6 months; tracing of defaulters who missed their subsequent vaccination doses; monitoring immunization performance as evidenced by use of monitoring chart; stock out of any vaccine for any duration; presence of trained EPI focal point; written feedback given by higher levels; interruption of immunization session for any reason; are care takers informed on key immunization messages after vaccinating their children?

We obtained the data on the independent and dependent variables from the WHO databases which are maintained in MS-Excel. We also used administrative coverage reports and the WHO database for data on supervision.

**Operational definitions:** DPT 3 coverage: proportion of under 1-year old infants vaccinated with three doses of DPT (Diphtheria, Pertussis, Tetanus) containing vaccine. A good immunization-performing zone was defined as a zone with at least 80% DPT 3 coverage and a poor performing zone was defined as one with less than 80% DPT 3 coverage. The Global Immunization Vision and Strategy (GIVS) and the national immunization Comprehensive Multi-Year Plan (cMYP) 2011-2015 documents aim to achieve at least 80% DPT 3 coverage in 90% of districts. Therefore the cut-off for categorizing zones in to good and poor performing zones is based on the national and global targets.

**Data analysis:** we checked both administrative and supervisory data for cleanliness, accuracy and missing values. We used MS Excel and Epi Info 3.5.3 [13] for cleaning and further data analysis. We performed descriptive analysis of variables and checked completeness and frequency of each variable. We recoded variables into yes/no and conducted bivariate analysis and explored the presence of association between the independent variables and zone immunization coverage. We decided to consider variable with small p-value (p<0.1) for multivariate analysis of possible prediction of immunization performance, adjusted for the presence of other variables.

## Results

We analyzed data on immunization management from 426 health facilities located in eight Regions and 53 zones. Of these 38 (71%) were categorized as good performing zones while15 (29%) were categorized as poor performing zones (Table 1).

**Table 1:** Regional distribution of supervised facilities in Ethiopia, 2014

Region	Frequency	Percent
Afar	24	5.6%
Amhara	81	19.0%
Benishangul Gumuz	8	1.9%
Gambella	18	4.2%
Oromia	163	38.3%
SNNPR	66	15.5%
Somali	45	10.6%
Tigray	21	4.9%
DPT-HIB-HepB3 performance of zones		
Good	38	79%
Poor	15	21%

Immunization sessions for catchment population were available in 396 (93%). Three hundred forty four (88%) and 166 (45.7%) facilities were supervised and given written feedback within the 6 months study period. There was no reported interruption of immunization sessions in 391 (94%) nor stock out of any vaccine in 264 (64%). Performance monitoring and defaulter tracing were observed in 207 (52%) and 217 (55%) health facilities respectively. Immunization sessions were observed in 116 (27.9%) of the facilities out of which 103 (88.8%) of vaccinators provided key EPI information to caretakers. Trained focal persons were available in-237 (56%) of the facilities (Table 2).

**Table 2:** Characteristics of supervised facilities by selected variables, Ethiopia, 2014

Characteristic	Number	Percent
**Are defaulters traced?**		
No	176	45%
Yes	217	55%
**Is the EPI focal person trained?**		
No	102	24%
within 2 years	237	56%
More than 2 years	82	19%
**Does the health facility provide feedback?**		
No	30	7%
Yes	396	93%
**Was the health facility supervised by woreda/zone?**		
Not supervised	47	12%
Supervised within 1-3 months	47	12%
Supervised within 3-6 months	294	76%
**Was there interruption of immunization service?**		
No	391	94%
Yes	25	6%
**Does the health facility monitor immunization performance**		
No monitoring chart	31	8%
Have monitoring chart but not updated	163	41%
Have monitoring chart and updated	207	52%
**Was there stock out of vaccine**		
No stock out	264	64%
Yes-stock out of DPT-HIB-HepB	8	2%
Yes-stock out of other vaccine	140	34%
**Has the health facility given written feedback from woreda/zone?**		
No	198	54.3%
Yes	166	45.7%
**Are care givers informed on key EPI messages?**		
No	13	11.2%
Yes	103	88.8%

Findings from bivariate analysis revealed strong association between DPT3 coverage and availability of immunization service in the facility [Odds Ratio (OR) =3.45. 95% Confidence Interval (CI) = 2.08,7.37, p value <0.01], written feedback [OR =5.72. 95% CI= 3.17, 10.32, p value <0.001], supervision of health facilities by woredas or zones [OR =3.92. 95% CI= 2.08 7.37, p value 0.001] and stock out of vaccine [OR =0.38. 95% CI= 0.24, 0.61, p value 0.001] (Table 3).

**Table 3:** Association of supervisory characteristics with DPT-HIB-HepB 3 coverage, Ethiopia, 2014

Variable	Low coverage # (Col %)	High coverage # (Col %)	OR (95% CI)	P value
**Are defaulters traced**				
Not traced	46 (50)	130 (43.2)	1.31(0.82-2.10)	0.25
Yes traced	46	171		
**Does HF provide immunization service**				
No	14 (14.9)	16(4.8)	3.45(1.62-7.38)	<0.01
Yes	80	316		
**HF supervised**				
No	23 (25.6)	24 (8.1)	3.92( 2.08-	0.001
Yes	67	274	7.37)	
**HF Monitor immunization performance**				
No	49 (53.8)	145(46.8)	1.33(0.83-2.12)	0.23
Yes	42	165		
**Any interruption of immunization session**				
No	88(97.8)	303(92.9)	3.34(0.77-	0.08
Yes	2	23	14.4)	
**Stock out of any vaccine**				
No	42 (46.2)	222 (69.2)	0.38(0.24-	0.001
Yes	49	99	0.61)	
**Available trained focal person**				
No	41(45.1)	143 (43.3)	1.07(0.67-1.71)	0.77
Yes	50	187		
**Written feedback given by the higher level**				
No	75(82.4)	123(45.1)	5.72(3.17-	<0.001
Yes	16	150	10.32)	
**Key EPI information provided to caregivers**				
No	4(11.4)	9(11.1)	1.03(0.82-2.10)	0.59
Yes	31	72		

Logistic regression analysis revealed that, based on our categorization of good performance, DPT3 coverage was 7.16 and 4.35 times greater respectively in zones that conducted supportive supervision within the preceding 3-6 months (95%CI=1.92, 26.26, p value 0.00) and provided written feedback to their health facilities (95%CI =2.27, 8.33, p value 0.00). The DPT3 coverage was 20.15 times greater in zones where the health facilities had immunization services (95%CI=2.24, 181.38, p value 0.01) and 4.1 times greater where health facilities monitored their immunization performance (95%CI =1.2, 14.05, p value 0.02). The DPT3 coverage was 0.44 times lesser in zones where health facilities had stock out of vaccines (OR 0.44 95%CI = 0.23-0.83, p value 0.01) (Table 4).

**Table 4:** Logistic regression analysis of variables with strong association as predictors for DPT-HIB-HepB3 performance, Ethiopia, 2014

Term	Odds Ratio	95% C.I.	S. E.	Z-Statistic	P-Value
Were health facilities supervised within 3-6 months(Yes /No*)	7.16	[1.92-26.6]	0.67	2.93	0.00
Were health facilities supervised within 3 months (Yes /No*)	4.15	[1.85-9.32]	0.41	3.45	0.00
Were health facilities providing immunization services (yes/No*)	20.15	[2.24-181.38]	1.12	2.68	0.01
Did health facilities monitored performance (Yes-not updated/No*)	3.80	[1.10-13.10]	0.6318	2.11	0.03
Did health facilities monitored performance (Yes-updated/No*)	4.10	[1.20-14.05]	0.63	2.25	0.02
Was there interruption of immunization session (Yes/No*)	6.38	[0.63-65.01]	1.18	1.56	0.11
Was there stock out of vaccines (Yes-Other vaccine stockout/No stockout*)	0.44	[0.23-0.83]	0.3223	-2.55	0.01
Was there stock out of vaccines (Yes-DPT-HIB-HepB stockout/No stockout*)	0.14	[0.02-0.97]	0.98	-1.99	0.04
Was there written feedback given by higher level (yes/No*)	4.35	[2.27-8.33]	0.33	4.44	0.00
CONSTANT	*	*	1.32	-3.50	0.00

## Discussion

In our analysis of factors associated with good performance of RI, we found availability of immunization sessions, supervision, written feedback, performance monitoring and stock out of vaccines to be strong predictors of immunization coverage in Ethiopia [9, 14–16]. Written feedback, supportive supervision, availability of immunization services in all health facilities and performance monitoring were associated with good performance. Stock out of vaccine was associated with poor performance[14, 15, 17]. In our logistic regression analysis, we also found that performance monitoring was associated with good immunization performance though this was not observed on bivariate analysis[18]. We also observed that stock out of vaccines other than DPT containing vaccines were associated with poor DPT3 coverage containing vaccine probably through interruption of session until all antigens are ready for immunization sessions [14, 19].

To our surprise, we did not observe any association between coverage and the following characteristics: provision of key EPI messages to care takers, availability of trained EPI focal person and interruption of session and defaulter tracking. We considered that the association between coverage and provision of key messages might have been affected due to observation of inadequate immunization sessions [18,20]. Generalization of our findings to other contexts may be limited by the following: we conducted cross-sectional study which may not be a preferred method for comparison analysis. However, we analyzed variables with p-value <0.1 and explored factors contributing to immunization performance. Data from the two city administrations (Addis Ababa and Dire Dawa) and Harari region was not available. However, data were available from the remaining regions and we included urban zones in our analysis.

## Conclusion

In conclusion, though we did not include all potential predictor variables in the study, we found that the way health facilities manage immunization programme and the supportive function of higher levels are predictors of immunization performance of zones in Ethiopia. We recommend that administrative health offices should provide regular written feedback and enforce provision of immunization service in all health facilities where there are adequate immunization system inputs. We further recommend that stock management should improve at all levels and the negative impact of stock out of vaccines other than pentavalent should be noted; therefore stock management should aim at improving availability of all vaccines at all times. We also recommend regular supervision of health facilities which provides the opportunity to identify and rectify gaps as they occur. We also recommend further study incorporating other potential variables and more settings to gather more evidence in Ethiopia.

### What is known about this topic

- While there is adequate information on the importance of conducting supportive supervision to health units and provision of feedback, little is documented about the contribution of such actions to improve immunization performance;
- Most studies focus on identifying challenges to immunization performance and try to forward recommendations to increase coverage by removing the identified challenges than focusing on identifying the positive factors that contribute to better performance;
- Little effort is made to understand why some areas perform well compared to the others while being under the same socio-economic and political environment.

### What this study adds

- This study gives a different perspective as to looking at the factors that contribute to immunization by trying to understand the positive attributes than focusing on challenges;
- This study also gives evidence that the actions of the higher levels such as supervision and written feedback when done regularly and properly can influence performance positively;
- The study provides evidence as to the contribution of health facility characteristics to immunization performance apart from the experience and characteristics of service providers and service users.

## Competing interests

The authors declare no competing interests. The views expressed in the perspective articles are those of the authors alone and do not necessarily represent the views, decisions or policies of the institutions with which they are affiliated and the position of World Health Organization.
